# Editorial: Signal transduction of plant organ senescence and cell death

**DOI:** 10.3389/fpls.2023.1172373

**Published:** 2023-03-28

**Authors:** Zhonghai Li, Ralf Oelmüller, Hongwei Guo, Ying Miao

**Affiliations:** ^1^ State Key Laboratory of Tree Genetics and Breeding, College of Biological Sciences and Technology, Beijing Forestry University, Beijing, China; ^2^ Matthias Schleiden Institute, Plant Physiology, Friedrich-Schiller-University Jena, Jena, Germany; ^3^ Key Laboratory of Molecular Design for Plant Cell Factory of Guangdong Higher Education Institutes, Department of Biology, Southern University of Science and Technology (SUSTech), Shenzhen, Guangdong, China; ^4^ Fujian Provincial Key Laboratory of Plant Functional Biology, Fujian Agriculture and Forestry University, Fuzhou, Fujian, China

**Keywords:** epigenetical regulation, lncRNA, protein phosphorylation, peptide hormones, macronutrient, leaf senescence

The senescence of plant organs, the final stage of organ development, is a form of programmed cell death (Miao and Zentgraf, 2007). It is characterized by the functional transition from nutrient assimilation to nutrient remobilization, which is crucial for plant fitness and affects crop yield, quality, and horticultural performance (Guo and Gan, 2005; Lim et al., 2007). Although it has been reported that leaf senescence impacts photosynthesis, nutrient mobilization, stress responses, and productivity (Guo et al., 2021), the contributions of a myriad of natural parameters, such as organ age, coordination between different regulatory pathways, source-sink relationships, nutrient remobilization, and anterograde/retrograde signal transduction during organ senescence, remain to be unraveled.

This Research Topic compiles a total of ten articles, four reviews, and six research studies, covering five topics: i) New discoveries in the multiple layers of regulation of leaf senescence; ii) Recent progress in the regulation of leaf senescence by classical and peptide hormones; iii) Novel signaling components regulating organ senescence; iv) New mechanisms for nutrient deficiency-induced leaf senescence; and v) Latest breakthroughs in leaf senescence research methods and techniques.

Leaf senescence is a systematic physiological process that involves several tiers of regulation, including at the level of chromatin remodeling, as well as at the transcriptional, translational, and post-translational levels, as revealed by multi-omics analyses. (Woo et al., 2013; Woo et al., 2019) Miao’s team reported that histone acetylation (H3K9ac) enrichment accompanied the transcriptional induction of senescence-associated genes (SAGs) during leaf senescence in Arabidopsis and rice (Huang et al., 2018; Zhang et al., 2022a; Huang et al., 2022). In Arabidopsis, histone deacetylase 15 (HDA15) interacting with an ssDNA-binding protein WHIRLY1 acts as a repressor of downstream gene transcription, leaf senescence, and flowering (Huang et al., 2022). Zareen et al. reported that histone deacetylase 9 (HDA9) and POWERDRESS (PWR) complex recruiting a transcriptional corepressor HIGH EXPRESSION OF OSMOTICALLY RESPONSIVE GENE15 (HOS15) acts as a positive regulator to promote leaf senescence upon aging and dark stress by repressing the histone acetylation at several gene loci, such as *NPX1*, *APG9*, and *WRKY57*. However, many unidentified mechanisms at the epigenetic modification level still need to be clarified.

Long non-coding RNAs (lncRNAs) are a type of non-coding RNAs that are emerging as hidden players in many biological processes (Statello et al., 2021). In this Research Topic, Kim et al. performed comprehensive analyses of RNA-seq data representing all leaf developmental stages to determine the genome-wide lncRNA landscape during Arabidopsis leaf aging, providing a valuable resource of age-related (AR) lncRNAs and proposing a potential gene regulatory network of leaf senescence that links the function of protein-coding mRNAs and AR-lncRNAs. Recent advances in the fields of non-coding RNAs, epigenetic modifications, and alternative splicing in the regulation of leaf senescence have all been reviewed (Guo et al., 2021; Zhang et al., 2021; Miryeganeh, 2022).

Protein phosphorylation/dephosphorylation plays a crucial role in the leaf senescence process. Controlled protein dephosphorylation by protein phosphatases is vital to containing the extent of senescence. Several protein phosphatases that positively or negatively influence the induction or progression of this process have been identified (Zhang and Gan, 2012; Xiao et al., 2015; Durian et al., 2020). Protein kinase, in turn, functions in signal transduction *via* the phosphorylation of downstream signaling components to activate the regulatory network (Ahmad and Guo, 2019). Miao et al. reported that MITOGEN-ACTIVATED PROTEIN KINASE (MAPK) KINASE KINASE1, MEKK1, can take a shortcut and directly phosphorate the WRKY53 protein as well as activate *WRKY53* gene expression and leaf senescence (Miao et al., 2007). Wu et al. show that MPK3 and MPK6, two Arabidopsis MAPKs, and their two upstream MAPK kinases, MKK4 and MKK5, act *via* the MKK4/5, MPK3/6, and MATRIX METALLOPROTEINASE (MMP) At2/At3 cascade as key regulators of leaf senescence. Yang et al. reviewed the recent progress in plant leaf senescence-related kinases and summarized the current understanding of the function of kinases in senescence signal perception and transduction.

The role of classic phytohormones, such as abscisic acid (ABA), ethylene, jasmonic acid (JA), salicylic acid (SA), brassinolide (BR), gibberellin (GA), and auxin indole-3-acetic acid (IAA) that function as important signaling molecules in plants and contribute to the onset and progression of leaf senescence, has been well documented (Guo et al., 2021). Increasing evidence now shows that peptide hormones CLAVATA3/ESR-RELATED (CLEs), Phytosulfokine (PSK), and INFLORESCENCE DEFICIENT IN ABSCISSION (IDA) or IDA-like (IDLs) peptides are also involved in the regulation of leaf senescence, expanding the repertoire of signaling molecules that control leaf senescence (Zhang et al., 2022b; Zhang et al., 2022c). In this issue, Guo et al. reported that the IDL6 peptide is a positive regulator of leaf senescence. Huang et al. presented recent advances in our understanding of leaf senescence regulation by classical and peptide hormones.

Organ senescence, a type of programmed cell death, leads to the massive retrieval of nutrients from senescing organs to the rest of the plant (Rogers, 2013; Schippers et al., 2015). In addition to carbohydrate and energy remobilization during leaf, petal, and seed senescence (Chrobok et al., 2016; Huang et al., 2020; Zhang et al., 2021; Zhu et al., 2022), many macronutrients, such as magnesium (Mg), iron (Fe), and nitrogen (N), also get recycled and channeled into essential cellular processes such as an extensive range of metabolic, regulatory, and structural activities (Guo et al., 2015; Guo et al., 2021). The deficiency or excess of these nutrients seriously affects plant growth and development (Shi et al., 2012; Tanoi and Kobayashi, 2015; Yang and Udvardi, 2018). For example, Mg as a constituent of magnesium porphyrin plays a role in retrograde signaling and ABA-induced senescence (Koussevitzky et al., 2007). In this issue, Kocourkova et al. showed that in *PHOSPHOLIPASE Dα1*-deficient mutant plants, *pldα1-1*, higher accumulation of ABA and JA, and impaired homeostasis of Mg, potassium, and phosphate were observed under high-Mg^2+^ conditions. Furthermore, high Mg^2+^ also led to an increase in starch and proline content in Arabidopsis plants. PLDα1 was concluded to act as a negative regulator of high-Mg^2+^-induced leaf senescence. Finally, in this article collection, Sakuraba et al. reviewed the current understanding of the molecular mechanisms associated with N starvation-induced leaf senescence.

Given that senescence is affected by numerous developmental and environmental signals, such as biotic and abiotic stresses (Guo et al., 2021), research on organ senescence requires systematic approaches and sophisticated experimental designs. Plant scientists are searching for rapid experimental systems to reveal the molecular regulatory mechanisms of leaf senescence controlled by multiple factors. Protoplasts are an effective experimental system for employing rapid and systematic cellular approaches to dissect gene function in Arabidopsis (Tyurin et al., 2020) and to perform genetic manipulation in crops (Ghogare et al., 2021). In this issue, Kim et al. established a transient gene expression assay in *Arabidopsis* protoplasts and validated this system by monitoring the differential expression of *LUCIFERASE*-based reporters driven by the promoters of SAGs (*SEN4-LUC* and *SAG12-LUC*) (Doan et al., 2022). This approach provides a valuable system for studying senescence at the cellular and molecular levels in various species.

This article collection is a testament to the notion that, even though impressive progress has been made in the identification and functional analysis of a large number of SAGs in plants, many urgent scientific questions remain in this field, such as when plant senescence is initiated or how senescence signals are transmitted between organelles, cells, tissues, and organs, as well as how to best address the molecular mechanisms underlying cell senescence. With the application of single-cell multi-omics analysis and gene-editing technologies, such as CRISPR/Cas9, the precise mechanisms governing cell senescence will be deciphered, and a wide variety of genome-modified stay-green crops will be developed and commercialized in the foreseeable future.

## Author contributions

ZL and YM prepared drafts of the manuscript, and RO and HG revised the manuscript. All authors have read and agreed to the published version of the manuscript.
